# Supplementary material for: ST-segment elevation myocardial infarction with non-obstructive coronary arteries: Score derivation for prediction based on a large national registry
Source: PLoS One. 2021 Aug 5;16(8):e0254427. doi: 10.1371/journal.pone.0254427 (PMC8341573; doi:10.1371/journal.pone.0254427)
Supplement: Attachment — Submitted filename: Responses to the Reviewers Comments .doc [file pone.0254427.s001.doc]

PONE-D-20-39776
ST-segment elevation myocardial infarction with non-obstructive coronary arteries: derivation of a score for prediction based on a large national registry
PLOS ONE

Dear Dr. Januszek,

Thank you for submitting your manuscript to PLOS ONE. After careful consideration, we feel that it has merit but does not fully meet PLOS ONE’s publication criteria as it currently stands. Therefore, we invite you to submit a revised version of the manuscript that addresses the points raised during the review process.


Please submit your revised manuscript by Feb 25 2021 11:59PM. If you will need more time than this to complete your revisions, please reply to this message or contact the journal office at plosone@plos.org. When you're ready to submit your revision, log on to https://www.editorialmanager.com/pone/ and select the 'Submissions Needing Revision' folder to locate your manuscript file.

Please include the following items when submitting your revised manuscript:

- A rebuttal letter that responds to each point raised by the academic editor and reviewer(s). You should upload this letter as a separate file labeled 'Response to Reviewers'.
- A marked-up copy of your manuscript that highlights changes made to the original version. You should upload this as a separate file labeled 'Revised Manuscript with Track Changes'.
- An unmarked version of your revised paper without tracked changes. You should upload this as a separate file labeled 'Manuscript'.

This was modified.

If you would like to make changes to your financial disclosure, please include your updated statement in your cover letter. Guidelines for resubmitting your figure files are available below the reviewer comments at the end of this letter.

If applicable, we recommend that you deposit your laboratory protocols in protocols.io to enhance the reproducibility of your results. Protocols.io assigns your protocol its own identifier (DOI) so that it can be cited independently in the future. For instructions see: [http://journals.plos.org/plosone/s/submission-guidelines#loc-laboratory-protocols](http://journals.plos.org/plosone/s/submission-guidelines#loc-laboratory-protocols)

We look forward to receiving your revised manuscript.

Kind regards,

Carmine Pizzi
Academic Editor
PLOS ONE

Journal requirements:

When submitting your revision, we need you to address these additional requirements.

1. Please ensure that your manuscript meets PLOS ONE's style requirements, including those for file naming. The PLOS ONE style templates can be found at

https://journals.plos.org/plosone/s/file?id=wjVg/PLOSOne_formatting_sample_main_body.pdf and

https://journals.plos.org/plosone/s/file?id=ba62/PLOSOne_formatting_sample_title_authors_affiliations.pdf

 This was modified.

2. You indicated that ethical approval was not necessary for your study. We understand that the framework for ethical oversight requirements for studies of this type may differ depending on the setting and we would appreciate some further clarification regarding your research. Could you please provide further details on why your study is exempt from the need for approval and confirmation from your institutional review board or research ethics committee (e.g., in the form of a letter or email correspondence) that ethics review was not necessary for this study? Please include a copy of the correspondence as an ""Other"" file.

Due to the fact that the study is conducted on data from the ORPKI register, where the data is entered without names, addresses, telephone numbers or the identification number of individual patients, the consent of the bioethical commission is not required for such research. In addition, one of the works from this database has already been published in the PLOS One Journal, and then, also such documents were not required, which has not changed in this respect.

Januszek R, Dziewierz A, Siudak Z, Rakowski T, Dudek D, Bartuś S. [Chronic obstructive pulmonary disease and periprocedural complications in patients undergoing percutaneous coronary interventions.](https://pubmed.ncbi.nlm.nih.gov/30273363/) PLoS One. 2018 Oct 1;13(10):e0204257.

3.In your Data Availability statement, you have not specified where the minimal data set underlying the results described in your manuscript can be found. PLOS defines a study's minimal data set as the underlying data used to reach the conclusions drawn in the manuscript and any additional data required to replicate the reported study findings in their entirety. All PLOS journals require that the minimal data set be made fully available. For more information about our data policy, please see http://journals.plos.org/plosone/s/data-availability.

 The data has been deposited at: [Home | OSF](https://osf.io/dashboard)/ DOI 10.17605/OSF.IO/MD7AT.

Upon re-submitting your revised manuscript, please upload your study’s minimal underlying data set as either Supporting Information files or to a stable, public repository and include the relevant URLs, DOIs, or accession numbers within your revised cover letter. For a list of acceptable repositories, please see http://journals.plos.org/plosone/s/data-availability#loc-recommended-repositories. Any potentially identifying patient information must be fully anonymized.

 The data has been deposited at: [Home | OSF](https://osf.io/dashboard)/ DOI 10.17605/OSF.IO/MD7AT.

Data included at the doi enable calculations of the following values included in the tables and figures:
> - The values behind the means, standard deviations and other measures reported
> - The values used to build graphs
> - The points extracted from images for analysis

Important: If there are ethical or legal restrictions to sharing your data publicly, please explain these restrictions in detail. Please see our guidelines for more information on what we consider unacceptable restrictions to publicly sharing data: http://journals.plos.org/plosone/s/data-availability#loc-unacceptable-data-access-restrictions. Note that it is not acceptable for the authors to be the sole named individuals responsible for ensuring data access.

Not applicable, simplified version of data has been deposited at : [Home | OSF](https://osf.io/dashboard)/ DOI 10.17605/OSF.IO/MD7AT.

We will update your Data Availability statement to reflect the information you provide in your cover letter.

Not applicable, simplified version of data has been deposited at: [Home | OSF](https://osf.io/dashboard)/ DOI 10.17605/OSF.IO/MD7AT.

4.Thank you for stating the following financial disclosure:

 "NO - The funders had no role in study design, data collection and analysis, decision to publish, or preparation of the manuscript."

At this time, please address the following queries:

a. Please clarify the sources of funding (financial or material support) for your study. List the grants or organizations that supported your study, including funding received from your institution.

The statement: “The funders had no role in tge study design, data collection, analysis, decision to publish, or preparation of the manuscript” has been added to the Cover Letter.

b. State what role the funders took in the study. If the funders had no role in your study, please state: “The funders had no role in study design, data collection and analysis, decision to publish, or preparation of the manuscript.”

The statement: “The funders had no role in tge study design, data collection, analysis, decision to publish, or preparation of the manuscript” has been added to the Cover Letter.

c. If any authors received a salary from any of your funders, please state which authors and which funders.

The statement: ”The authors received no specific funding for this work” has been added to the Cover Letter.

d. If you did not receive any funding for this study, please state: “The authors received no specific funding for this work.”

The statement: ”The authors received no specific funding for this work” has been added to the Cover Letter.

Please include your amended statements within your cover letter; we will change the online submission form on your behalf.

This was modified according to the recommendations specified above.

5.We noticed you have some minor occurrence of overlapping text with the following previous publication(s), which needs to be addressed:

https://www.dovepress.com/characteristics-of-patients-with-myocardial-infarction-with-nonobstruc-peer-reviewed-article-VHRM
https://www.mdpi.com/2077-0383/9/11/3610/html

The text that needs to be addressed involves sections of the Introduction and Discussion.

This was rephrased and modified.

In your revision ensure you cite all your sources (including your own works), and quote or rephrase any duplicated text outside the methods section. Further consideration is dependent on these concerns being addressed."

This was corrected.

Reviewers' comments:

Reviewer #1: In this paper Jędrychowska et al. create a MINOCA STEMI predictive score using the regression model, in order to distinguish, in STEMI group, MINOCA patients from MI-CAD patients; younger age, female gender, no history of prior CABG, no history of smoking, no arterial hypertension, presence of COPD, no treatment with 3rd generation P2Y12, no direct transport to hospital, Killip class I or II, no history of diabetes and greater body mass at admission were considered significant predictors of MINOCA among STEMI patients qualified for urgent coronary angiography and used to develop the score. The coefficients calculated were used to construct the predictive model in the form of a nomogram. The model showed that patients who scored more than 600 points had a 19% probability of MINOCA, and for patients scoring more than 650 points, the probability of MINOCA was 71%.

The study is interesting and focuses on a relatively new diagnostic problem such as the MINOCA patients. Nevertheless, I have some major concerns:

1) MINOCA diagnosis --> according to ESC Guidelines? --> how do you exclude the cases of non-ischemic troponin elevation? These patients are not MINOCA (3° point of ESC Position Paper).

According to the guidelines, patients with a working diagnosis of STEMI do not require, and even it is not advisable, troponin assessment, should it delay revascularization (<120 min). Thus, the diagnosis of STEMI is sufficient with the EKG and clinical symptoms during first medical contact.

Ibanez B, James S, Agewall S, Antunes MJ, Bucciarelli-Ducci C, Bueno H, Caforio ALP, Crea F, Goudevenos JA, Halvorsen S, Hindricks G, Kastrati A, Lenzen MJ, Prescott E, Roffi M, Valgimigli M, Varenhorst C, Vranckx P, Widimský P; ESC Scientific Document Group. [2017 ESC Guidelines for the management of acute myocardial infarction in patients presenting with ST-segment elevation: The Task Force for the management of acute myocardial infarction in patients presenting with ST-segment elevation of the European Society of Cardiology (ESC).](https://pubmed.ncbi.nlm.nih.gov/28886621/) Eur Heart J. 2018; 39: 119-177.

2) There are no data on troponin. How was STEMI diagnosed?

According to the ESC guidelines, the diagnosis of STEMI can be made on the basis of the ECG itself and clinical symptoms during the first medical contact.

Ibanez B, James S, Agewall S, Antunes MJ, Bucciarelli-Ducci C, Bueno H, Caforio ALP, Crea F, Goudevenos JA, Halvorsen S, Hindricks G, Kastrati A, Lenzen MJ, Prescott E, Roffi M, Valgimigli M, Varenhorst C, Vranckx P, Widimský P; ESC Scientific Document Group. [2017 ESC Guidelines for the management of acute myocardial infarction in patients presenting with ST-segment elevation: The Task Force for the management of acute myocardial infarction in patients presenting with ST-segment elevation of the European Society of Cardiology (ESC).](https://pubmed.ncbi.nlm.nih.gov/28886621/) Eur Heart J. 2018; 39: 119-177.

3) Table 1: The majority of MINOCA patients with prior AMI, had also prior PCI --> so they supposed to have a significative CAD. How these patients could be classified as MINOCA?

Previous PCI does not exclude STEMI and MINOCA. STEMI MINOCA can be diagnosed both in patients following PCI and in those after CABG in the past - in other words, a positive history of advanced atherosclerosis does not exclude MINOCA.

4) Table 1: 67 patients classified as MINOCA had a previous CABG. The same as before, these patients had significative CAD. I suppose they are not MINOCA.

Here we have a similar justification as in the case of the point describede above.

5) Table 2: “Considering patients from the MINOCA group, 71.57% had significant stenoses while 28.43% no visible atherosclerosis”. If these patients had significant CAD, why they are called MINOCA?

In Table 2, it is shown that in the MINOCA group, 71.57% of patients had atherosclerotic lesions in their coronary arteries, but th significance was not confirmed. However, there is no information stating that 71.57% of the MINOCA group had significant stenosis.

6) What was the cause of MINOCA? Is there any information for the prevalence of plaque disruption, coronary artery spasm, coronary thromboembolism, coronary dissection?

Unfortunately, the data provided in the present publication come from a registry that collects only periprocedural data and is supplemented by operators of catheterisation laboratories. Therefore, we do not have data on the final etiology of MINOCA. It is widely known that to establish the etiology of MINOCA in a large percentage of cases, even many weeks after the initial incident, is conditioned by the need to perform additional tests, such as a thrombophilic test package, magnetic resonance imaging of the heart or other additional diagnostic tests. However, when it comes to finding coronary spasm or dissection, including spontaneous coronary dissection, we have such data, but due to the large percentage of missing data, we have refrained from including this in statistical calculations due to the high probability of bias and drawing erroneous conclusions. This was added to the “Strengths and limitations” section.

7) It is sufficient to approximate the numbers in the tables to the first decimal. Tables will be more readable.

This was modified in Tables 1-3, however, we have left the data presented so far in Table 4-5, due to the need to maintain differences for individual confidence intervals.

8) 45% of MINOCA are in DAPT. In these patients, was DAPT therapy administered even without DES implantation?

The results of the analysed data, which are presented in the current publication, refer to the pre-hospital period, often directly pre-hospital, therefore, it is not possible to assess the impact on prognosis regarding the amount of antiplatelet drugs in the follow-up period. Certainly, in some patients, DAPT therapy is associated with recent PCI or acute coronary syndrome in recent months, but it is not possible to estimate the percentage based on the available data collected in the registry. This was clarified in the “Discussion section”.

9) The score assigned to each variable is not clear.

The graph allows to show a scale for all main effects in the regression formula. Covariate distributions are superimposed on nomogram scales.

10) The main purpose is understanding how distinguish a priori a patient with MINOCA and possibly not to perform a coronary study. I believe that the results are absolutely not suitable for reaching this conclusion. Even if the score is > 650, the probability that it is a MINOCA is about 71%; therefore, it means that about 30% of patients are not MINOCA, actually; I don't think with these numbers it is possible to decide not to perform a CAG to a patient when it is recognized that an early PTCA can improve the prognosis. The criteria for performing a CAG as soon as possible (in STEMI) have been formulated precisely because they admit a low sensitivity and therefore provide for the possibility of having false positives with patients with free coronary arteries.

We definitely agree with the raised doubts, however, the fact that this approach is currently in place does not change the fact that some of the patients with STEMI do not have significant lesions in the coronary arteries, and in the future, it may be tempting to create such an algorithm that would with an almost 100% likelihood rule out patients with STEMI who do not require coronary angiography. Such possibilities can also be obtained from an accurate image scan that could be performed in fast mode. The purpose of this article is to spark a discussion on this topic. This was additionally clarified in the “Conclusions” section.

11) AUC 0.7 --> It seems to me little bit low.

This is true, but please see the standard error of 0.000046, which is very small indicating very precise predictive value.

Reviewer #2: M. Jędrychowska and coworkers studied the main clinical and angiography differences in a large population of STEMI in both obstructive acute myocardial infarction and MINOCA patients. Data were collected between January 2014 and December 2019, and were selected from 1,177,218 patients who underwent coronary angiography. The authors aimed to create a tool facilitating a rapid separation of the MINOCA patients from the entire group of obstructive STEMI ones.
Finally, they evaluated a score able to address and separate MINOCA from obstructive AMI patients hospitalized due to MI with ST segment elevation. The model showed that patients who scored more than 600 points had a 19% probability of MINOCA, and for patients scoring more than 650 points, the probability of MINOCA was 71%. The other end of the MINOCA probability scale was marginal for patients who scored less than 500 points (< .2%).
The strengths of this study are:
- First, the large population of the study. Indeed they evaluated more than 1 million patients from the Polish National Registry of Percutaneous Coronary Interventions (ORPKI). They selected a broad sample of STEMI underwent PCI and MINOCA with ST-segment elevation (more than 5600 ones). Respect to the current literature on MINOCA, this is a very large population though from a retrospective registry.
- They collected only ST-segment acute myocardial infarction. This is a specific group of patients, especially in the MINOCA, which is worth studying due to higher mortality, particularly intra-hospital.
- The idea of a model able to distinguish STEMI obstructive AMI vs STEMI MINOCA could be very useful in clinical practice due to the subsequent diagnostic and therapeutic implications.
- I appreciate the table 2 in which they showed the coronary angiography and procedural indices.

However, there are some limitations of this study. The major pitfalls are:
- MINOCA patients are a heterogeneous entity. The current literature considered as MINOCA only coronary ischemic causes of acute myocardial infarction without obstructive coronary arteries. In our clinical practice, we have to exclude non-cardiac causes of troponin surge but also cardiac conditions like tako-tsubo syndrome or acute myocarditis.
In this work, the MINOCA population is quite heterogeneous and seemed quite unselected. They had not well-established inclusion and exclusion criteria for MINOCA diagnosis. Moreover, what were the main causes of MINOCA? Did they use cardiac magnetic resonance or other secondary diagnostic tools to address the final MINOCA diagnosis, as the recent guidelines recommend?

The current database covers only the pre-hospital period and is limited to the stay in at catheterisation unit, thus, we are not able to analyse the final diagnosis, due to the fact that diagnostic tests which are necessary to make a final diagnosis, such as cardiac magnetic resonance or examinations for thrombophilia, are performed after coronary angiography. The presented analysis includes patients prior to laboratory tests (cTn assessment), diagnosed with STEMI according to current ESC guidelines (based on ECG and clinical symptoms) during First Medical Contact (FMC).

Ibanez B, James S, Agewall S, Antunes MJ, Bucciarelli-Ducci C, Bueno H, Caforio ALP, Crea F, Goudevenos JA, Halvorsen S, Hindricks G, Kastrati A, Lenzen MJ, Prescott E, Roffi M, Valgimigli M, Varenhorst C, Vranckx P, Widimský P; ESC Scientific Document Group. [2017 ESC Guidelines for the management of acute myocardial infarction in patients presenting with ST-segment elevation: The Task Force for the management of acute myocardial infarction in patients presenting with ST-segment elevation of the European Society of Cardiology (ESC).](https://pubmed.ncbi.nlm.nih.gov/28886621/) Eur Heart J. 2018; 39: 119-177.

- I think that the best clinical utility of the diagnostic model, created in this work, is to rule out the probability of MINOCA diagnosis. In fact, if the score is less than 500 points the probability of MINOCA is quite low < .2%. However, this model could not help the clinicians to avoid an urgent coronary angiography in patients with a suspected STEMI, even if the probability of obstructive CAD is low.

Yes, we definitely agree with this assumption, however the main aim of the current study was to raise a discussion, and in the future, make an attempt to create a scheme that reduces the number of unnecessarily performed invasive coronary angiography in patients with acute STEMI.

In the paper, the authors should explain more the clinical role of this predictive score (eg: in patients with high MINOCA probability, it’s useful to use more diagnostic tools, like left ventriculography during angiography or intravascular imaging, and to design a specific treatment in this population).

Thank you for that valuable remark. We added more information on this subject in the “Conclusions” section.

- It could be useful to create a predictive model with only pre-angiography variables, eg exclude P2Y12 inhibitors and add other variables like the presence of typical angina. This could be more useful for the clinicians as explained above.

Yes, we definitely agree, and thank you for this valuable remark, however the registry did not contain such information. As such, we are not able to include this data in the current analysis.

Minor limitation:
- I suggest a more careful revision of the English language in the text

The text has been extensively and carefully proof-edited by a native speaker, professional translator and proofeditor (**AmE Native** **Katarzyna Smith-Nowak,** ul. Miejscowa 8, 30-499 Kraków, e-mail: [english.native123@gmail.com](mailto:english.native123@gmail.com), Phone: 0048 505 990 391, NIP: PL6792878140), to ensure proper cohesion, coherence and style of the manuscript, while maintaining its substantive content. The general readability for an English-speaking audience should be appropriate, the flow of the text ensured.

Below, please find a point-by-point list regarding the types of alterations/items checked in the text:

- Extensive stylistic editing

- Eradication of repetitions and redundancies as well as spelling mistakes

- Correction of grammatical number

- Correction of spacing, punctuation

- Exchanging colloquial linguistic items for more formal ones

- Maintaining consistency of spelling

- Correction of preposition usage

- Correction of article usage

- Maintaining linguistic consistency within the text

-Maintaining consistent usage of numerical format in data presentation

- Corrections regarding parts of speech

- Maintaining cohesion and coherence

- Changes in word order

We hope that the text in present form is in accordance with and meets all applicable standards of your renown Journal.
