# Supplementary material for: ST-segment elevation myocardial infarction with non-obstructive coronary arteries: Score derivation for prediction based on a large national registry
Source: PLoS One. 2021 Aug 5;16(8):e0254427. doi: 10.1371/journal.pone.0254427 (PMC8341573; doi:10.1371/journal.pone.0254427)
Supplement: Attachment — Submitted filename: Response to Reviewers-R2.docx [file pone.0254427.s002.docx]

**Responses to the reviewers comments**

Reviewer #3: This work intended to design a score for predicting MINOCA among STEMI patients in the Polish national registry of PCIs. They analyzed 124,663 pPCI treated individuals and 5,695 individuals with STEMI and MINOCA. The results showed the significant difference between patients with MINOCA and those in the MI-CAD group based on the proposed MINOCA score.

1. Please make the table self-explained. The notation needs to be noted. For example, 120 [60 ÷ 330]. What does it mean in bracket?

This was corrected.

2. Need to precisely provide the information how the score was constructed in method section.

3. Validation is required to comment on the model performance. It’s unclear how the authors evaluate the performance of the score. It seems that the same sample was used. If so, it would lead to inflated results for the performance. If not, detail information should be provided.

Response to comments 2 and 3. The ‘Methods’ section was improved, detailed methods for nomogram creation were presented. The bootstrap validation model was implemented, with the bias corrected index used to evaluate the model’s prognostic performance.

Reviewer #4: 

The key concerns is in relation to the MINOCA definition and diagnosis.

The 4th Universal Definition of AMI states that MINOCA is:
MI patients with no angiographic obstructive CAD (> 50% diameter stenosis in a major epicardial vessel) AND, MINOCA, like the diagnosis of MI, indicates that there is an ischaemic mechanism responsible for the myocyte injury (i.e., non-ischaemic causes such as myocarditis have been
excluded).

This was clarified in the ‘Methods’ and ‘Limitations” sections.

There are challenges of the use or misuse of the term MINOCA since the term should be exclusively reserved for those patients with confirmed MI and thus who have undertaken key investigations such as cardiac MRI.

This was clarified in the ’Methods’ section and highlighted in the ‘Limitations’ section.

There is very limited data on such patients and institutions have limited availability of additional diagnostic testing. As such the presented cohort in this study, like many others, is a heterogenous cohort of ‘suspected MINOCA’. This should be acknowledged upfront.

This was modified in the ‘Methods’ section.

The authors should adopt this language of ‘suspected MINOCA’ in their manuscript and refer to the 4th Universal Definition in their limitations.

This was modified in the ‘Methods’ section.

The term ‘predictive score for MINOCA’ or ‘MINOCA predictive score’ should not be used as this is misleading due to the heterogeneity in the cohort.

This was modified according to the suggestions.

The introduction should also be clarified as other non-coronary conditions such as myocarditis do not cause MINOCA but rather mimic MINOCA.

This sentence has been rephrased, as recommended by the Reviewer.

The scientific reasoning provided for the need for such a predictive score requires careful consideration also. The authors state that ‘coronary angiography is only an exclusive tool’ and whilst there is no relevance of primary PCI in MINOCA, the diagnostic angiogram in STEMI is still relevant to identify other underlying conditions such as Takotsubo or spontaneous coronary artery dissection.
Such a tool being used to ‘triage’ patients away from urgent angiography inappropriately may cause harm.
The aim in the introduction should be re-worded, in particular the phrase ‘reach a diagnosis’ should be removed.

This was corrected.

The results show that the MINOCA patients are significantly younger compared to MICAD.
Where subsequent analyses age-adjusted to reflect this important difference, in particular, for CVD risk factors which are known to increase with ag?

The final multiple regression model that was used to create the nomogram includes age as a covariate, hence, the results are already age-adjusted.

In relation to coronary angiography data, the authors state 71.57% of MINOCA had significant stenoses. This is confusing.

This was corrected.

Since MINOCA patients, by definition, have non-obstructive CAD, I assume the authors refer to plaques less than 50%?

This was clarified in the ‘Methods’ section.

Can the authors please clarify this and confirm that the MINOCA and MICAD populations were defined based on the 50% stenosis threshold. Were these thresholds ascertained by the angiography reports and/or operating physicians?

This was modified.

The nomogram description (“in the form of a nomogram, that is, a graphical representation of the relative impact of each prognostic factor within the global model”) in the results should be moved to the Methods.

This was corrected and this paragraph has been moved to the chapter on ‘Methods’.

In the conclusion, the main aims should be carefully re-considered
“The main aim of this publication was to spark a discussion, and in the future, try to create a
scheme reducing the number of unnecessarily performed invasive coronary angiographies in
patients with acute STEMI.”

It may be difficult to find clinical acceptance for a need to reduce unnecessary angiograms in STEMI.
However, the authors make an excellent point that a potential use of such predictive algorithms is for pre-paring the operator for a possible MINOCA diagnosis and thus for additional testing. This is a key point that should be further emphasised as a major issue in current MINOCA management is the lack of additional diagnostic testing. The discussion of a possible MINOCA diagnosis would also help prepare patients who are often left with no understanding of their presentation and discharged without a diagnosis.
Thus, the use of predictive algorithms in MINOCA has a place but the derivation population must be defined appropriately, and the focus of the tool is used to improve management and communication in the health care process.

This was corrected.
