# Supplementary material for: ST-segment elevation myocardial infarction with non-obstructive coronary arteries: Score derivation for prediction based on a large national registry
Source: PLoS One. 2021 Aug 5;16(8):e0254427. doi: 10.1371/journal.pone.0254427 (PMC8341573; doi:10.1371/journal.pone.0254427)
Supplement: Attachment — Submitted filename: Response to Reviewers.docx [file pone.0254427.s003.docx]

**Responses to the reviewers comments**

PONE-D-20-39776R2
ST-segment elevation myocardial infarction with non-obstructive coronary arteries: score derivation for prediction based on a large national registry
PLOS ONE

Dear Dr. Januszek,

Thank you for submitting your manuscript to PLOS ONE. After careful consideration, we feel that it has merit but does not fully meet PLOS ONE’s publication criteria as it currently stands. Therefore, we invite you to submit a revised version of the manuscript that addresses the following points: 

Please submit your revised manuscript by Aug 07 2021 11:59PM. If you will need more time than this to complete your revisions, please reply to this message or contact the journal office at plosone@plos.org. When you're ready to submit your revision, log on to https://www.editorialmanager.com/pone/ and select the 'Submissions Needing Revision' folder to locate your manuscript file.

Please include the following items when submitting your revised manuscript:

- A rebuttal letter that responds to each point raised by the academic editor and reviewer(s). You should upload this letter as a separate file labeled 'Response to Reviewers'.
- A marked-up copy of your manuscript that highlights changes made to the original version. You should upload this as a separate file labeled 'Revised Manuscript with Track Changes'.
- An unmarked version of your revised paper without tracked changes. You should upload this as a separate file labeled 'Manuscript'

Please review your reference list to ensure that it is complete and correct. If you have cited papers that have been retracted, please include the rationale for doing so in the manuscript text, or remove these references and replace them with relevant current references. Any changes to the reference list should be mentioned in the rebuttal letter that accompanies your revised manuscript. If you need to cite a retracted article, indicate the article’s retracted status in the References list and also include a citation and full reference for the retraction notice.

Additional Editor Comments:

Though review articles are appropriately used as overview citations for broad scientific topics or ideas, most citations, especially those focusing on previously published concepts or results, should be of original research papers. The community also values the accurate assignment of credit and precedence for scientific discoveries. As so, please include in your revision references of larger studies, reporting contemporary cohorts in order to inform the audience on the heterogeneity of patients with MINOCA, specifically:

- Introduction, page  1, lines 2-3:  Circ Cardiovasc Qual Outcomes . 2017 Dec;10(12):e003443. doi: 10.1161/CIRCOUTCOMES.116.003443.

This reference has been added.

- Introduction page 1, lines 8-12: reference to this recently published original paper regarding the mechanism and etiologies of MINOCA should be added instead of the review paper-reference # 4: Circulation. 2021;143:624–640 doi: 10.1161/CIRCULATIONAHA.120.052008

The reference: Niccoli G, Scalone G, Crea F. Acute myocardial infarction with no obstructive coronary atherosclerosis: mechanisms and management. Eur Heart J. 2015; 36: 475-481. <https://doi.org/10.1093/eurheartj/ehu469> PMID: 25526726 has been removed and instead of that suggested reference was added

- Introduction, page  2, lines 2-3:  Circ Cardiovasc Qual Outcomes . 2017 Dec;10(12):e003443. doi: 10.1161/CIRCOUTCOMES.116.003443 and Journal of the American Heart Association. 2020;9:e017235 doi: 10.1161/JAHA.120.017235

This has been modified, and suggested referenses were addedd

- Discussion in regard with the following statement” What is interesting, in studies on the use of DAPT in MINOCA patients, it is shown that therapy is not only of no benefit, but may be detrimental in this group of patients [20,21]”
  - Reference # 20 could be misleading for the audience and should be revised accordingly. The study was an underpowered single-center study and as such is not able to detect the possible benefit or harm of therapeutic regimes in the MINOCA population. References of larger robust studies should be reported instead see Circulation.  2017 Apr 18;135(16):1481-1489. doi: 10.1161/CIRCULATIONAHA.116.026336.

This reference (20) has been replaced by suggested citation.

- Reference # 21 refers to an abstract. The full manuscript recently published, should be reported instead: Heart. 2021 Jan 27;heartjnl-2020-318045. doi: 10.1136/heartjnl-2020-318045. PMID: 33504513.

This reference (21) has been replaced by suggested citation.

- Additionally, with regard to the above-mentioned statement, it should be noted that, so far, possible harm associated with DAPT is mainly related to higher major bleeding rates, given also the higher incidence of MINOCA in women. In this regard the post-hoc analysis of the OASIS-7 trial (Heart. 2021 Jan 27;heartjnl-2020-318045. doi: 10.1136/heartjnl-2020-318045.) very nicely inform us about the possible harm related to intensified dosing strategy. Therefore, bleeding risk should be mentioned briefly.

This was corrected.

We appreciate the Reviewers’ and Editors’ efforts very much and hope that the answers provided below, as well as the manuscript revisions are satisfactory and sufficient to clear a path to the final acceptance of our contribution to the *PLOS One Journal*.

Additionally, as required by the Journal, we wanted to conclude that the funders had no role in the study design, data collection and analysis, decision to publish, or preparation of the manuscript, and that the authors received no specific funding for this work.

I am looking forward to hearing from you again.

Yours faithfully,

Rafał Januszek MD, Ph.D.

Department of Cardiology and Cardiovascular Interventions

ul. Jakubowskiego 2, 30-688 Kraków

jaanraf@interia.pl

Phone: +48-12-400-22-50; Fax: +48-12-400-22-67
